# Prolonged duration of response in lenvatinib responders with thyroid cancer

**DOI:** 10.1530/ERC-18-0049

**Published:** 2018-04-17

**Authors:** Andrew G Gianoukakis, Corina E Dutcus, Nicolas Batty, Matthew Guo, Mahadi Baig

**Affiliations:** 1 Los Angeles Biomedical Research Institute and Division of Endocrinology and MetabolismDepartment of Medicine at Harbor-UCLA Medical Center, Torrance, California, USA; 2 David Geffen School of MedicineUniversity of California – Los Angeles, Los Angeles, California, USA; 3 Eisai Inc.Woodcliff Lake, New Jersey, USA

**Keywords:** lenvatinib, radioiodine-refractory differentiated thyroid carcinoma, duration of response, SELECT

## Abstract

We present an updated analysis of lenvatinib in radioiodine-refractory differentiated thyroid cancer (RR-DTC) with new duration of response (DOR) data unavailable for the primary analysis. In this randomized, double-blind, multicenter, placebo-controlled phase 3 study, patients ≥18 years old with measurable, pathologically confirmed RR-DTC with independent radiologic confirmation of disease progression within the previous 13 months were randomized 2:1 to oral lenvatinib 24 mg/day or placebo. The main outcome measures for this analysis are DOR and progression-free survival (PFS). The median DOR for all lenvatinib responders (patients with complete or partial responses; objective response rate: 60.2%; 95% confidence interval (CI) 54.2–66.1) was 30.0 months (95% CI 18.4–36.7) and was generally similar across subgroups. DOR was shorter in patients with greater disease burden and with brain and liver metastases. Updated median PFS was longer in the overall lenvatinib group vs placebo (19.4 vs 3.7 months; hazard ratio (HR) 0.24; 99% CI 0.17–0.35; nominal *P* < 0.0001). In lenvatinib responders, median PFS was 33.1 months (95% CI 27.8–44.6) vs 7.9 months (95% CI 5.8–10.7) in non-responders. The median DOR of 30.0 months seen with patients who achieved complete or partial responses with lenvatinib (60.2%) demonstrates that lenvatinib responders can have prolonged, durable and clinically meaningful responses. Prolonged PFS (33.1 months) was also observed in these lenvatinib responders.

## Introduction

Differentiated thyroid cancer (DTC) is the most common type of thyroid cancer in the United States, where papillary and follicular thyroid carcinomas account for up to 94% of all thyroid carcinoma cases (Busaidy & Cabanillas 2012). The standard treatment for most patients receiving diagnoses of DTC is surgery followed by administration of radioactive iodine (Cooper* et al.* 2009). However, approximately 10–15% of cancers become refractory to radioiodine treatment, which is then referred to as radioiodine-refractory DTC (RR-DTC) (Busaidy & Cabanillas 2012, Pacini* et al.* 2012). For these cases, the life expectancy is 3–6 years and the 10-year survival rate is 10% from the time of metastatic detection (Durante* et al.* 2006, Pacini* et al.* 2012, Xing* et al.* 2013). Patients with RR-DTC have few treatment options and typically require alternative therapies (Pacini* et al.* 2012).

Tyrosine kinase inhibitor (TKI) therapy is a recently approved option for patients with RR-DTC. The first targeted agent shown to improve progression-free survival (PFS) in patients was the multikinase inhibitor, sorafenib (Brose* et al.* 2014), approved for use in patients with RR-DTC in 2013 by the US Food and Drug Administration (FDA) (Worden 2014). More recently, based on the results of the randomized, double-blind, multicenter, phase 3 Study of (E7080) Lenvatinib in Differentiated Cancer of the Thyroid (SELECT), the FDA approved lenvatinib for the treatment of locally recurrent or metastatic, progressive RR-DTC (Schlumberger* et al.* 2015). Lenvatinib is an oral, multikinase inhibitor of vascular endothelial growth factor (VEGF) receptor (VEGFR) 1–3, fibroblast growth factor receptor (FGFR) 1–4, platelet-derived growth factor receptor alpha (PDGFRα), ret proto-oncogene (RET) and stem cell factor receptor (KIT) (Matsui* et al.* 2008*a*,*b*, Okamoto* et al.* 2013, Tohyama* et al.* 2014, Yamamoto* et al.* 2014). In contrast to sorafenib, lenvatinib targets FGFR in addition to VEGFR, which is thought to be important for preventing the development of resistance to TKI therapies as the FGFR pathway offers an intracellular alternative to the VEGFR pathway (Laursen* et al.* 2016).

In the primary analysis of SELECT, lenvatinib was shown to significantly prolong PFS compared with placebo (18.3 vs 3.6 months; hazard ratio (HR) 0.21; 99% confidence interval (CI) 0.14–0.31; *P* < 0.001) (Schlumberger* et al.* 2015). At the time of the primary analysis, the median duration of overall response (DOR) had not been reached. Here, we report updated analyses of lenvatinib efficacy in SELECT with an emphasis on DOR.

## Materials and methods

### SELECT

The primary analysis of SELECT, a phase 3, randomized, placebo-controlled study, was previously reported (Schlumberger* et al.* 2015). Eligible patients were ≥18 years of age and had measurable, pathologically confirmed DTC, evidence of radioiodine-refractory disease, and independently reviewed radiologic evidence of progression within the previous 13 months. Patients were permitted to have received up to 1 prior treatment with a TKI. Patients were randomly assigned 2:1 to receive 24 mg of oral lenvatinib daily or placebo until disease progression, development of unacceptable toxicity or withdrawal of consent. The data cutoff for the primary analysis was November 15, 2013, after which the study continued with an open-label phase in which patients in the lenvatinib group could remain on therapy and those in the placebo group with progressive disease could choose to receive lenvatinib treatment. Patients in the placebo group who did not choose to receive treatment with lenvatinib (*n* = 22) in the open-label phase of the study were followed for survival. All patients enrolled in SELECT provided written informed consent. The study protocol was approved by the relevant institutional review bodies, and the study was conducted in accordance with the Declaration of Helsinki and local laws.

### Efficacy analyses

The data cutoff for this updated analysis was September 1, 2016. Tumors used in this updated analysis were assessed by clinical trial investigators. Responders were defined as patients who had a complete response (CR) or partial response (PR) as their best overall response per Response Evaluation Criteria in Solid Tumors v1.1. DOR was examined for lenvatinib-treated patients who had PR or CR overall and by patient subgroup (age, sex, tumor subtype, baseline disease burden, baseline Eastern Cooperative Oncology Group performance status, metastasis site or prior VEGF therapy). Exploratory efficacy endpoints included objective response rate (ORR), disease control rate (DCR) and clinical benefit rate (CBR). ORR was defined as the proportion of patients with best overall response of CR or PR. DCR was identified as the proportion of patients with a best overall response of CR or PR or stable disease (SD; achieved ≥7 weeks after Day 1 of the open-label treatment period). CBR was defined as the proportion of subjects who had a best overall response of CR or PR or durable SD (of duration ≥23 weeks).

In SELECT, the primary endpoint was PFS, which was assessed overall, including patients who did or did not respond to treatment. PFS is defined as the time from randomization until either objective tumor progression or death. Median PFS was estimated and plotted using the Kaplan–Meier method, and 95% CIs were constructed with a generalized Brookmeyer and Crowley method. HRs are estimated from a Cox proportional hazards model stratified by randomization factors. For overall PFS, the HR is expressed for lenvatinib and placebo, and for responder analysis, HR is expressed for responders and non-responders.

## Results

### Median duration of response of 30 months in responders

This updated analysis showed that patients who responded to lenvatinib (achieved CR or PR) could have prolonged, durable responses. The median DOR for all patients who responded to lenvatinib treatment was 30.0 months (95% CI, 18.4−36.7). A summary of the median DOR for all responders and by patient subgroup is shown in Table 1. Median DOR in lenvatinib-treated patients was generally similar by patient subgroup, but appeared to be shorter in patients with greater disease burden (tumor size ≤35 mm: 44.3 months; 35–60 mm: 27.5 months; 60–92 mm: 18.0 months; >92 mm: 15.7 months), patients with liver metastasis (yes: 15.7 months; no: 30.5 months) and patients with brain metastasis (yes: 9.3 months; no: 30.5 months). Median DOR was similar between responders who had received one prior VEGF therapy (29.9 months) and those who had not received prior VEGFR therapy (30.0 months).

**Table 1:** Median DOR for the lenvatinib treatment group in all responders and by subgroup.

Subgroup	*n*	Median DOR; lenvatinib treatment group; months (95% CI)
All responders	157	30.0 (18.4−36.7)
Age (years)		
≤65	104	27.5 (14.7−36.7)
>65	53	31.3 (18.4−43.5)
Sex		
Male	73	30.0 (16.8−43.5)
Female	84	27.3 (16.8−43.3)
Baseline diseases burden (mm)		
≤35	37	44.3 (30.5−NE)
35–60	45	27.5 (12.9−45.7)
60–92	38	18.0 (11.0−35.0)
>92	37	15.7 (11.1−35.2)
Bone metastasis only		
Yes	1	NE (NE−NE)
No	156	29.9 (18.4−36.7)
Lung metastasis		
Yes	141	29.9 (17.5−37.8)
No	16	34.0 (7.4−NE)
Liver metastasis		
Yes	24	15.7 (3.7−NE)
No	133	30.5 (22.2−41.4)
Brain metastasis		
Yes	5	9.3 (0.9−13.8)
No	152	30.5 (22.2−41.4)
Lymph node target lesions		
Yes	75	27.2 (12.9−35.2)
No	82	30.5 (22.2−NE)
Prior VEGF therapy		
Yes	40	29.9 (7.5−45.7)
No	117	30.0 (18.4−43.3)
Baseline tumor subtype		
Papillary	99	29.9 (16.8−43.3)
Follicular	58	30.0 (15.7−45.7)
Baseline ECOG PS		
0	102	31.3 (18.4−43.5)
1	52	27.5 (13.3−36.7)
>1	3	11.1 (0.9−11.1)

Updated data, cutoff: 1 September 2016.

CI, confidence interval; DOR, duration of response; ECOG PS, Eastern Cooperative Oncology Group performance status; NE, not evaluable; VEGF, vascular endothelial growth factor.

### Updated progression-free survival

**Figure 1:**
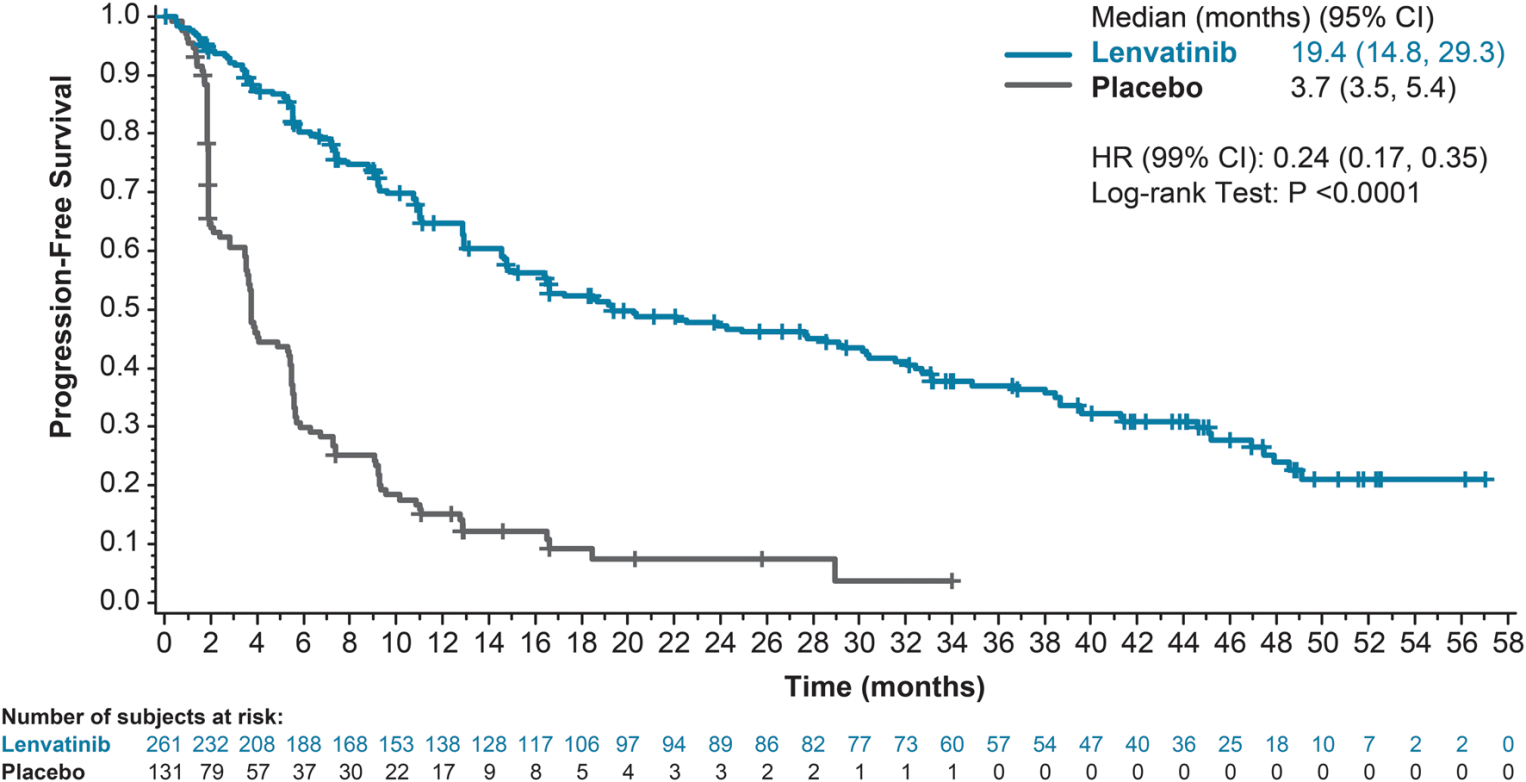
Kaplan–Meier estimate of progression-free survival by treatment. CI, confidence interval; HR, hazard ratio.

In SELECT, 261 (male: 125, female: 136) patients were enrolled to receive lenvatinib and 131 (male: 75, female: 56) patients received placebo treatment. The updated analysis showed a prolonged median PFS of 19.4 months in the lenvatinib group compared with 3.7 months in the placebo group (HR 0.24; 99% CI, 0.17−0.35; nominal *P* < 0.0001; Fig. 1). As of the updated data cutoff, 80.8% of lenvatinib-treated and 9.9% of placebo-treated patients from SELECT experienced grade ≥3 treatment-related treatment-emergent adverse events, and no new treatment-related deaths had occurred.

### Median progression-free survival of 33.1 months in responders

**Figure 2:**
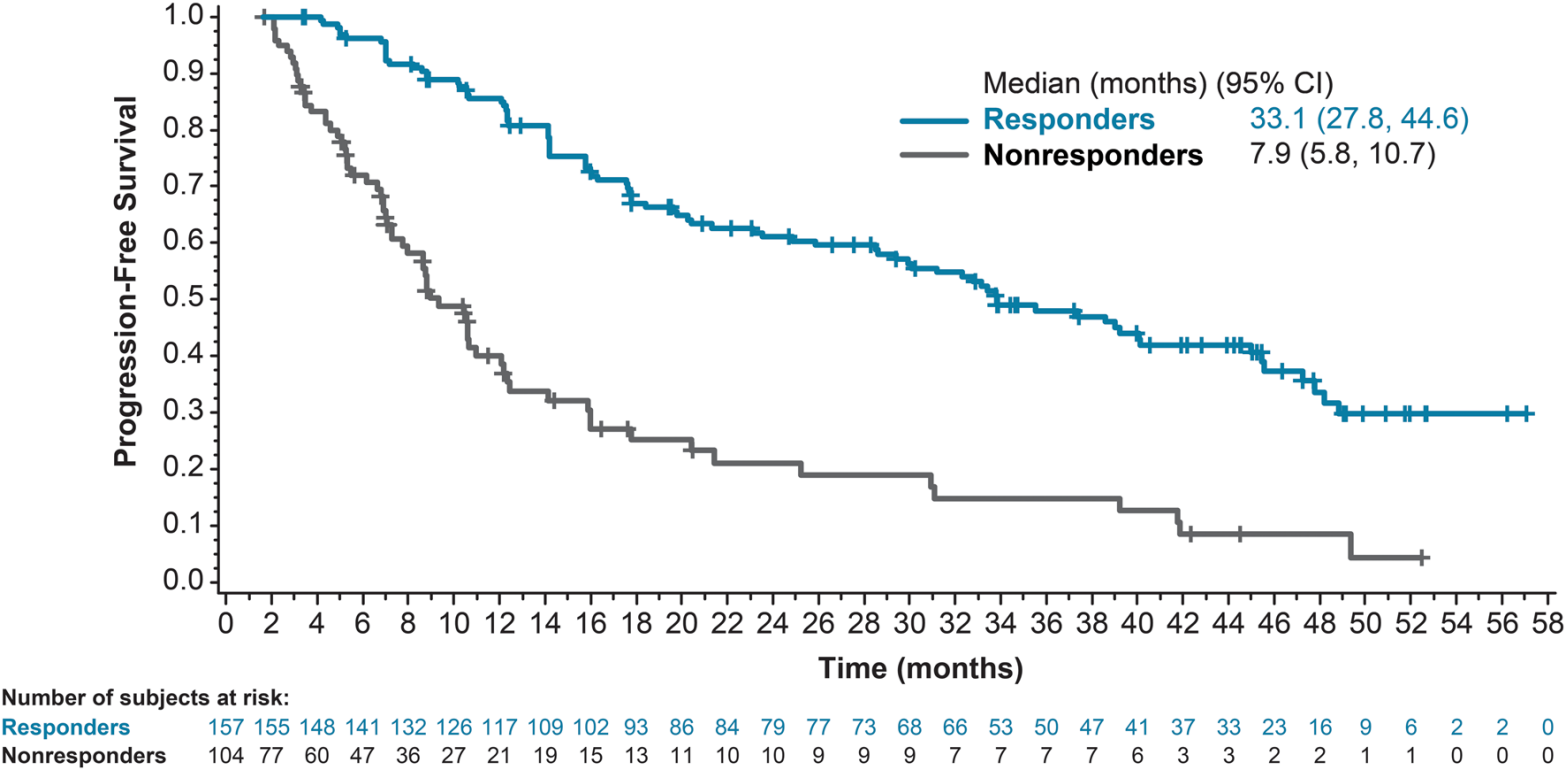
Kaplan–Meier estimate of progression-free survival for responders and non-responders. CI, confidence interval.

In the lenvatinib-treated group, the median PFS in patients who demonstrated a CR or PR was 33.1 months (95% CI, 27.8−44.6, Fig. 2), whereas in patients who did not show CR or PR, the median PFS was 7.9 months (95% CI, 5.8−10.7, Fig. 2).

The tumor response according to investigator assessment for this updated analysis is summarized in Table 2. The ORR was 60.2% (95% CI, 54.2−66.1) for lenvatinib-treated patients compared with 2.3% (95% CI, 0.0−4.9) for placebo-treated patients. Of note, since the primary analysis, 1 additional patient achieved a CR in both treatment groups. The DCR was 90.4% in the lenvatinib group compared with 61.1% in the placebo group (*P* < 0.0001), and the CBR was 82.0% for lenvatinib and 41.2% for placebo. Median time to first objective response was 3.5 months (95% CI, 1.9−3.7 months) in the lenvatinib group as evaluated by investigator assessment.

**Table 2:** Summary of tumor response per investigator assessment.

Parameter	Lenvatinib (*n* = 261)	Placebo (*n* = 131)
Best overall response, *n* (%)		
CR	5 (1.9)	1 (0.8)
PR	152 (58.2)	2 (1.5)
SD	79 (30.3)	77 (58.8)
Durable SD	57 (21.8)	51 (38.9)
PD	10 (3.8)	45 (34.4)
NE	2 (0.8)	2 (1.5)
Unknown	13 (5.0)	4 (3.1)
Objective response rate, *n* (%)	157 (60.2)	3 (2.3)
95% CI	54.2−66.1	0.0−4.9
Median time to first objective response, months (95% CI)	3.5 (1.9−3.7)	9.4 (1.8−11.0)
DCR, *n* (%)	236 (90.4)	80 (61.1)
95% CI	86.9−94.0	52.7−69.4
CBR, *n* (%)	214 (82.0)	54 (41.2)
95% CI	77.3−86.7	32.8−49.7
Median duration of SD, months (95% CI)	9.6 (7.6−14.8)	5.7 (5.5−7.4)

Updated data, cutoff: 1 September 2016.

CBR, clinical benefit rate; CI, confidence interval; CR, complete response; DCR, disease control rate; NE, not evaluable; PD, progressive disease; PR, partial response; SD, stable disease.

## Discussion

The durability of the responses observed measured by median DOR could not be estimated in the original analysis of lenvatinib in patients with RR-DTC from SELECT (Schlumberger* et al.* 2015). However, this updated analysis showed that patients who responded to lenvatinib continued to have prolonged, durable responses, lasting a median of 30.0 months. This prolonged DOR was observed across several patient subgroups and was not influenced by age, sex or tumor subtype. However, some interesting variations by subgroup were observed; for example, the median DOR was inversely correlated with a smaller disease burden. Importantly, the DOR was similar among patients who had prior anti-VEGF therapy (25%, *n* = 40) and those who did not, which demonstrates the effectiveness of treatment with lenvatinib. Differences in median DOR reported in some subgroups should be explored more fully in adequately designed and powered studies.

This updated data analysis also confirmed that lenvatinib is associated with prolonged PFS compared with placebo (median 19.4 months vs 3.7 months; HR 0.24; 99% CI, 0.17–0.35; nominal *P* < 0.0001), a similar PFS benefit as observed in the primary analysis of the trial (18.3 vs 3.6 months). However, notably, the median PFS in those patients who respond to lenvatinib treatment with a complete or partial decrease in tumor size was prolonged to 33.1 months, further emphasizing the promising efficacy demonstrated by lenvatinib in this patient population.

The only other agent approved by the FDA for RR-DTC is sorafenib. The development of resistance to sorafenib is widely observed in patients with thyroid cancer who initially experienced a PR or SD, and, therefore, a plan for alternative treatments is required for these patients (Pitoia & Jerkovich 2016). In this context, the durable response exhibited by patients in SELECT who received lenvatinib treatment, including those having failed prior anti-VEGF therapy, is especially important. The prolonged PFS and DOR observed with lenvatinib treatment may be due, in part, to lenvatinib’s inhibition of multiple intracellular signaling targets not targeted by other VEGF inhibitors, including FGFR (St Bernard* et al.* 2005, Laursen* et al.* 2016). Activation of the FGFR pathway has been implicated in the development of resistance to VEGF-targeted therapies (Dieci* et al.* 2013), and, therefore, it is possible that lenvatinib-mediated inhibition of FGFRs 1‒4 may play a role in the extended DOR exhibited by patients who responded to lenvatinib in SELECT.

In the primary analysis of SELECT, almost all patients in the lenvatinib treatment group experienced a treatment-related adverse event (Schlumberger* et al.* 2015), although most adverse events could be managed with dose modifications or medical therapy. We note that after 3 years of follow-up, the percentage of patients who experienced grade ≥3 lenvatinib-related adverse events increased by less than 5%, from 75.9% in the primary analysis to 80.8% in this analysis, with no new treatment-related deaths reported. This is consistent with a previous analysis of adverse events in SELECT, which concluded that most adverse events occurred early in the course of treatment (Haddad* et al.* 2017). This is important in the context of the prolonged DOR data showing that ongoing treatment with lenvatinib in those patients who demonstrate an initial response can result in a prolonged response to treatment. Thus, judicious management of adverse events in patients receiving lenvatinib long term is an especially important consideration for clinicians.

This analysis was limited by the lack of quality-of-life assessments. This prevents a complete evaluation of the benefits of prolonged lenvatinib treatment. Therefore, future studies of lenvatinib in this patient population should include quality-of-life assessments.

In conclusion, this updated analysis reinforced that lenvatinib treatment prolonged PFS compared with placebo in patients from SELECT with RR-DTC. Importantly, patients responding to lenvatinib demonstrated a prolonged PFS and DOR, suggesting that treatment with lenvatinib did not result in the same level of resistance as observed for some other TKIs. In addition, maintaining lenvatinib treatment by carefully managing adverse events can lead to a prolonged, durable response in 60.2% of patients. Further investigation is warranted to explore the considerations that must be made by clinicians treating patients with RR-DTC.

## Declaration of interest

Andrew G Gianoukakis reports grants and nonfinancial support from Eisai Inc. during the study. Corina E Dutcus and Matthew Guo are employees of Eisai, and Nicolas Batty and Mahadi Baig are former employees of Eisai.

## Funding

This work was supported by Eisai Inc., Woodcliff Lake, NJ, USA.
